# “When the climate changes, we feel it first”: Gendered perceptions of environmental change and women’s health in Pakistan

**DOI:** 10.1177/17455057261455288

**Published:** 2026-07-07

**Authors:** Abdul Rehman Nawaz, Iosif Botetzagias, Gulnaz Anjum

**Affiliations:** 1 47797 Deakin University, School of Humanities and Social Sciences, Melbourne, Australia; 2 68998 University of the Aegean, Department of Environment, Mytilene, Greece; 3 6305 University of Oslo, Department of Psychology, Oslo, Norway

**Keywords:** agricultural livelihoods, climate justice, climate change adaptation, faith-based framing, gendered perceptions, migration, Q methodology, structural vulnerability, women’s health

## Abstract

**Background:**

Rural women in climate-vulnerable regions face intersecting health risks from environmental stressors, gendered labor roles, and limited access to health services. Understanding how men and women perceive climate change is critical for designing interventions that protect women’s health and well-being.

**Objectives:**

To examine gendered perspectives on climate change among smallholder farmers in rural Pakistan and explore how these perspectives influence health-related risks and adaptive capacity, with a focus on implications for women’s health.

**Design:**

This study employed a cross-sectional Q-methodology design to identify shared and divergent viewpoints within and between male and female participant groups.

**Methods:**

Thirty participants (10 women and 20 men) from South Punjab, Pakistan, provided written informed consent. They sorted 48 statements into a forced quasi-normal distribution from “most disagree” to “most agree.” These individual sorts were analyzed, through PCA and varimax rotation, for identifying shared perspectives (‘Factors’ in Q terminology) separately for men and women. The qualitative interpretation of the factors was based on most (dis-) agreed upon, distinguishing and consensus statements.

**Results:**

Women’s factors, such as *Structural Health Vulnerability* and *Fatalistic Resilience*, emphasized infrastructural deficits, funding gaps, and health risks to food systems and livestock, linking adaptation capacity to systemic reforms. Men’s factors, including *Optimistic Techno-Adaptation* and *Economically Worried*, *Techno-Faithful*, displayed greater confidence in technological and market-driven solutions, though often overlooking structural constraints.

**Conclusion:**

Gendered framings of climate change reveal distinct implications for women’s health in rural Pakistan. Women’s perspectives emphasize systemic barriers that heighten vulnerability, underscoring the need for interventions that integrate climate adaptation with women’s health programs. Addressing faith-based framings, economic insecurity, and infrastructure gaps can improve the design of culturally resonant, gender-sensitive health interventions.

## Plain language summary

This study looks at how women and men farmers in rural Pakistan understand climate change and what they believe is needed to protect health, especially women farmers’ health. We spoke with smallholder farmers in South Punjab and asked them to rank statements about climate change, its causes, effects, and possible responses, allowing us to see where views overlap and where they differ. Women often described climate change as part of daily life (hotter days, shifting rainfall, sick animals, rising costs, and more pressure on food and care work) and highlighted barriers to adaptation such as poor roads and clinics, slow or missing support, and limited access to timely information, with many linking these problems directly to risks for mothers and children. Men showed a wider mix of views: some placed hope in technology and markets, others felt climate change is in God’s hands or that current impacts are manageable; these perspectives were less likely to focus on the structural barriers raised by women. Faith mattered for both groups, providing strength and meaning for some, but reducing the urgency to act for others. For policy and practice, climate plans should be designed with women, not just for them, and should invest in local health services, nutrition support, early warning systems, and usable information while respecting cultural and faith perspectives and improving access to resources and decision-making. By addressing both daily needs and broader system barriers, communities can build fairer and healthier paths to climate resilience.

## Graphical abstract

**Graphical Abstract:**
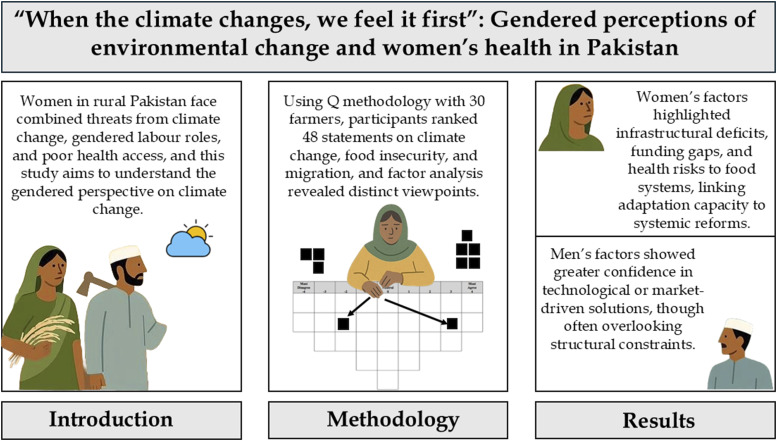

## Introduction

Feminist scholars have long emphasized that climate change (CC) goes beyond biophysical exposure to environmental hazards and is fundamentally shaped by structural marginalization.^1,2^ In many parts of the low- and middle-income countries (LMICs), women occupy a paradoxical position: they are simultaneously among the most vulnerable to climate impacts and the most active in sustaining everyday life - often at high cost to their own health and wellbeing.^3^ Their contributions, knowledge, and lived experiences remain systematically sidelined in dominant climate narratives, institutions and policy frameworks.^4,5^ This structural entanglement is known as “Green Patriarchy”^6^ referring to the systemic dominance of male-led institutions in climate governance that marginalize the knowledge and agency of women and gender-diverse groups.^7^ This is particularly evident in agrarian communities with Pakistan being no exception, where gendered roles in agriculture, caregiving, and migration intersect with limited land rights, unequal access to information, and exclusion from formal decision-making.^8^ The health consequences of this structural exclusion also remain substantially undertheorized, as the cumulative physical and mental health risks borne by women due to climate change receive insufficient attention in both research and policy.^9^

In Pakistan’s agrarian economy, rising temperatures, prolonged droughts, glacial melting, erratic monsoons, and floods increasingly disrupt the livelihoods of smallholder farmers.^10^ Climate Risk Index, in its report ‘*Who suffers most from the extreme weather events?’* ranks Pakistan 1st for the most vulnerable country to climate change.^11^ These climatic events disproportionately affect poor, marginalized, and vulnerable groups in society, with women being the most affected.^12–15^ Women constitute 68% of agricultural labour (as compared to 28% men) in Pakistan,^16^ however, their roles remain largely unrecognized in formal adaptation frameworks.^13,17^ Climate-related disruptions to food systems, water availability, and agricultural productivity disproportionately expose women to malnutrition, physical exhaustion, and heightened cardiometabolic risk.^18^ Under the current climate stress, the dual burden of agricultural and domestic labour has intensified, as women, especially in the Global South, travel longer distances for scarce water collection, contributing to chronic musculoskeletal injuries and physical exhaustion.^19^ Emerging evidence suggests that rising temperatures are also linked to adverse maternal outcomes, including preterm birth, low birth weight, and gestational hypertension.^20,21^ These risks are far greater where climate-induced flooding disrupts healthcare infrastructure and limits access to maternal services.

Climate change-related health risks are also linked to how farmers, especially women, experience, interpret, and respond to climate change in their everyday lives, as their domestic and societal roles simultaneously heighten their exposure to climate threats and shape their understanding of those risks.^9^ Despite bearing these risks and marginalization, women farmers demonstrate remarkable autonomy and resilience through everyday strategies rooted in faith, communal knowledge, and embodied labour. Yet, mainstream climate policy does not take gender perspectives into account, especially in studies led by men.^22^ Similarly, dominant frameworks tend to universalize adaptation as a rational, technical response, obscuring the moral, cultural, and spiritual dimensions that shape how women perceive and respond to environmental stress.^23–25^ In the present study, we investigate how farmers, both men and women, perceive climate change. We try to investigate attributions related to its causes, consequences, and adaptation pathways through the lens of locally situated epistemologies shaped by faith, marginality, and structural inequality.^26^

### Climate change, migration, and women’s health

A rapidly growing body of research has begun to examine the linkages between climate change, human migration, and population health^27–29^ and these linkages have gendered implications.^30^ Climate change-related events have undermined global household food security and eroded women’s health, agency and adaptive capacity.^9,31^ In rural Pakistan, many women engage in subsistence farming and also manage unpaid reproductive labour. These dual burdens are exacerbated by climate-related variabilities, which not only reduce crop yields but also increase the frequency of male out-migration, leaving women to shoulder agricultural responsibilities without institutional support and recognition.^12^ Where male out-migration occur, remittances can partially offset these health disadvantages, however, this benefit remains contingent on household wealth and women’s own education levels.^32^ Climate adaptation in such contexts is not only a technical challenge but also a gendered political issue which is deeply inter-linked with histories of exclusion and patterns of systemic neglect.^33,34^ Despite women’s crucial roles in agricultural production, they are structurally disadvantaged by limited land ownership, restricted mobility, and gendered norms that confine decision-making to men.^13^

Human migration, being an adaptation^35^ and survival strategy,^36^ due to climatic variabilities and disasters has also become important in gender discussion.^37–39^ In Pakistan, the floods of 2022 displaced tens of millions of people, destroyed agricultural land, and submerged rural communities.^40^ During these disasters, women disproportionately face the emotional, mental, physical, and social burdens of forced displacement coupled with increased domestic workloads, unpaid agriculture work, vulnerability to harassment, and restricted mobility^12^ which make them more vulnerable to acquiring infectious diseases.^41^ Multiple case studies document how climate-induced migration (CIM) affects socioeconomic conditions, disrupts healthcare access, and impacts women’s health, especially maternal health,^42^ particularly among poor, rural communities, pregnant women, and female headed households.^29,43–47^

The health consequences of climate-related migration on women are disproportionate and overlooked. However, growing evidence has linked exposure to extreme heat, floods and air pollution during migration to preterm birth, low birth weight, stillbirth, and congenital anomalies, while displaced women’s limited access to prenatal care and safe birthing facilities, unplanned pregnancies, contribute to higher rates of maternal mortality, preeclampsia, and gestational hypertension.^48–53^ In flood-affected areas, contaminated ponds, wells, and rivers increases the prevalence of cholera, typhoid fever, and diarrheal diseases. As a result, rural women who live the close proximity to the water supplies bear a disproportionate burden, given their roles in water collection, leading to urinary tract infections, injuries and other health complications.^54,55^ These physical risks are intensified by psychological distress, depressive disorder, and generalized anxiety among displaced women.^56,57^ Climate-induced migration acts as a threat multiplier for women’s health, where it exacerbates existing gender inequalities and compounds their vulnerability to poor health outcomes that extend well beyond the period of displacement itself.

### Faith, situated climate knowledge and adaptation

Women in Pakistan frame climate change not solely through scientific paradigms but through spiritual and moral frameworks. Faith-based explanations of droughts and floods (e.g., as tests from God or signs of collective moral decline) are often dismissed by technocratic planners as irrational and fatalistic. However, scholars argue that such perspectives constitute legitimate, situated forms of knowledge.^23,33,58^ In rural Pakistan, Islamic beliefs often structure everyday life and offer resources for psychological resilience, collective ethics, and adaptive action.^59^

Women farmers may simultaneously invoke divine agency and engage in pragmatic responses such as crop diversification or water conservation. This hybridity challenges the binary between “traditional” belief and “modern” adaptation. Farming related adaptation decisions are often embedded in moral economies shaped by trust, kinship, and spiritual commitments, not just material constraints (also see,^60^). Like any identity, women’s gender identity is greatly influenced by their traditional beliefs. Anjum and colleagues show how Pakistani women negotiate cultural continuity and religious belief as part of identity reconstruction both at home and in exile.^61–63^ These findings are crucial for reframing how faith-based discourses of climate are interpreted: not as signs of disengagement, but as cultural idioms through which women make sense of vulnerability and agency.

Recent research shows how access to safe water, nutrition, and healthcare declines in such contexts, exacerbating heat-related illnesses and maternal health risks.^64^ Current climate policies continue to overlook these specific gendered vulnerabilities.^65^ The care-related adaptation nexus must therefore be analyzed through a feminist lens that captures not only physical care burdens but also the emotional, epistemic, and structural burdens women experience.^9,66^ The present study is an effort to investigate how faith shape women’s perceptions of climate risks and their perceived ability to adapt to climate change.

### Theoretical framing: Intersectionality and decolonial feminist perspective

Intersectionality provides a foundational lens for this study. As Crenshaw^67^ theorized that marginalized identities are shaped not by additive oppressions but by interlocking systems (e.g., patriarchy, class, caste, and religion) that define access, risk, and voice. For Pakistani women farmers, these structures create compounded vulnerabilities: as landless workers, caregivers, Muslims, and often as widows or abandoned spouses in migration-affected households.^13^ This intersectional condition is visible in the way women interpret their roles in climate adaptation. Decisions about whether to migrate, switch crops, or seek institutional support are not merely pragmatic choices but are constrained by community norms, religious obligations, and social expectations around shame, honor (izzat), and family duty.^61–63^ There is further intersection of gender and rurality in the context of Pakistan.^68^ The decolonial feminist lens critiques the universalizing tendencies of Western climate discourse, especially in its treatment of women in the Global South as either helpless victims or hyper-resilient agents.^69^ In Pakistani contexts, this dualism erases the structural and historical forces, such as colonial land laws, Islamization policies, and neoliberal austerity, that shape women’s everyday lives.^61^

### Current study

In this study, we approach women’s climate narratives as acts of epistemic resistance. Women farmers’ understandings of climate change, whether framed through religion, memory, or embodied labour, are not inferior alternatives to “scientific” discourse but are legitimate ways of knowing rooted in experience, spirituality, and history. The objective of this study is to systematically examine gendered perspectives on climate change among smallholder farmers in rural Pakistan, with particular attention to their implications for women’s health and wellbeing. Specifically, the research question guiding this work is: How do women and men farmers perceive and prioritize the risks, causes, and adaptive responses to climate change, and in what ways do these gendered framings shape vulnerabilities and adaptive capacities relevant to women’s health?

## Methodology

We applied Q-methodology, a mixed-methods approach that combines qualitative and quantitative techniques to systematically explore shared viewpoints within and between groups. Q-methodology is particularly suited for examining subjective perspectives on complex issues^70,71^ such as climate change, as it allows for the identification of latent patterns of agreement and disagreement across participants. Q methodology was used to elicit and compare gendered framings of climate change among smallholder farmers in rural Punjab, Pakistan.

### Study site

**Figure 1.**
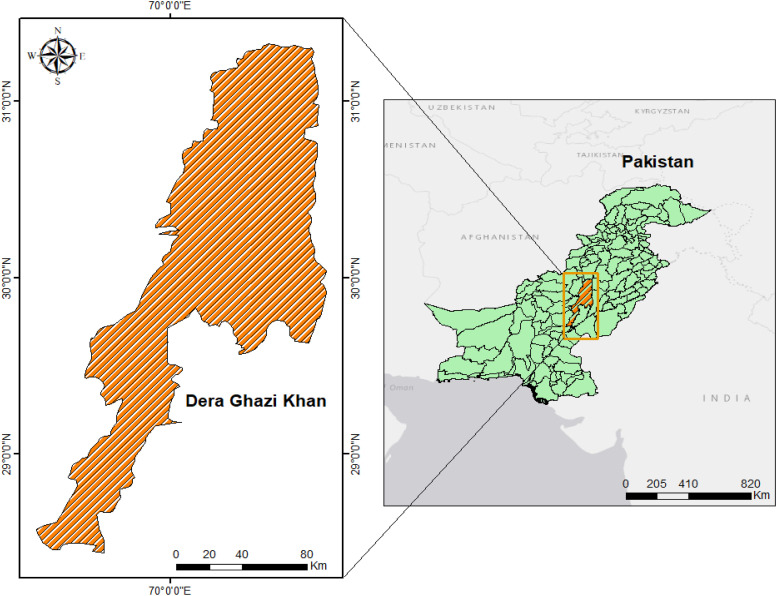
Map of the study area.

The study was conducted in Dera Ghazi Khan (DGK) district, southern Punjab, Pakistan. DGK is a region highly vulnerable to the impacts of climate change, including extreme heat, floods, and droughts (see study area map in Figure 1). Agriculture is the primary livelihood source of communities living in DGK, and climate variability directly affects their food security, water availability, and household health. The region is prone to floods and has experienced over 25 major floods since 1950.^72^ In 2022, floods damaged 342 villages, inundated 80 union councils, and affected nearly 699,502 people.^73^ Situated between the Koh-Sulaiman Range and the Indus River plains, DGK is also prone to destructive monsoon-fed hill torrents.^74^ Agriculture, livestock, and poultry-key livelihoods in the district-are highly sensitive to climate extremes, with recent events reducing household resilience and increasing vulnerability.^75,76^ Further information about the study area is provided in the supplementary material.

Figure 1. Map of the study area. Location of Dera Ghazi Khan district in southern Punjab, Pakistan, where Q-sorts were conducted. Source: Developed by the authors using ArcGIS Pro.

### Application of Q-methodology and data sources

Q methodology is a mixed-methods approach for systematically investigating human subjectivity, how individuals think, feel, and prioritize on a given topic.^77^ Unlike traditional variable-centered (“R”) methods, Q methodology is person-centered approach, identifying holistic configurations of viewpoints through factor analysis.^70,78^ It is particularly suited to complex issues such as climate change, as it captures participants’ perspectives from their own frame of Reference 71 and can be implemented effectively with small, purposively selected samples.^79^ Q methodology has been applied across disciplines, including health care^80^ and climate change adaptation research^81,82^ and women’s health,^83,84^ making it well suited for examining gendered framings of climate change and their implications for women’s health. This study was conducted and reported in accordance with the Standards for Reporting Qualitative Research (SRQR) guideline.^85^ A summary of Q methodology advantages is provided in supplementary material Table S1.

Following established procedures,^79,86^ the ‘concourse’ for this study (see supplementary material) was developed using a ready-made approach, drawing on validated statements from studies conducted^81^ in Bangladesh and^82^ Iran. The original 76 statements were organized into five thematic areas: (1) causal beliefs, (2) causes, (3) perceived risks, (4) impacts, and (5) adaptation (following^81^). From this pool, 48 statements were retained to serve as the Q set (i.e. the statements to be presented to the study’s participants). Statements were screened for clarity, cultural relevance, and non-redundancy before inclusion, and their selection was based on thematic breadth, clarity, and cultural relevance. On occasion they were refined for comprehensibility and translated into Urdu for field use. The Q-set list, categorized by thematic area, is provided in supplementary Table S2.

### Inclusion and exclusion criteria

To be eligible, participants were required to be at least 18 years of age and to have completed matriculation-level education, so that they could independently read, think, and rank the Q-sorts without external assistance. Participants were also required to have direct farming experience within their household, either as primary farmers or as active contributors to household agricultural activities. All participants were found to have direct experience with farming and agricultural livelihoods.

### Participant recruitment

Thirty smallholder farmers were purposively selected (10 women; 20 men), representing diversity in age, education level, landholding size, and village location (see Table 1 for participant characteristics). Q methodology uses purposive sampling (a P-set) to ensure that all relevant perspectives are represented, regardless of their frequency in the general population^70^ and does not require power analysis, as it is not designed to test hypotheses. The gender imbalance reflects under representation of women as formal landholders in the study area and the cultural constraints affecting women’s participation in agricultural decision-making. As is standard in Q methodology, data saturation was not pursued in the conventional sense. Instead, adequacy was ensured through the comprehensiveness of the Q sample in representing the full range of discourse and the purposive selection of participants to reflect diverse perspectives.

**Table 1.** Sociodemographic features of study’s participants (n=30).

Variables/Areas	n	Percentage
Gender		
Female	10	33.3
Male	20	66.7
Age		
Below 30	19	63.3
31-40	8	26.7
41-50	3	10
Above 50	0	0
Education		
Bachelors	7	23.3
FA/FSc	7	23.3
MA/MSc/MS	5	16.7
Matric	10	33.3
MBBS/LLB	1	3.3
Villages		
Basti Wadani	1	3.3
Choti Zareen	3	10
Gadai	6	20
Peer Fateh Shah	3	10
Pir Adil	16	53.3
Sakhi Sarwar	1	3.3

### Procedure

All participants were verbally briefed in Urdu and then provided written informed consent through the Q-TIP web platform before the Q-sorting began. Each participant completed a ‘Q-sort’, i.e., individually ranking the Q-set’s 48 statements along a forced quasi-normal distribution grid ranging from “-4: most disagree” to “+4: most agree”, with ‘0’ as the middle point (see Figure S2 in Supplementary material). Prior to sorting, participants were verbally briefed on the study objectives and provided written informed consent via the web Q-sort platform. The Q-sorting process began with participants reading all statements, followed by three stages: (1) initial categorisation into agree, disagree, or neutral; (2) placement into the forced distribution grid (Figure S2, illustrating the Q-sort distribution grid); and (3) final adjustments until the ranking reflected their views. Sorting sessions were conducted individually and face-to-face, from April 8, 2025 to April 24, 2025 with the help of a research assistant to ensure comprehension, particularly for participants with limited literacy. The sorting process was carried out in Urdu, using statements translated and back-translated from English to ensure accuracy and cultural appropriateness, through Q-TIP,^1^ a free, web-based tool developed by researchers from the University of Wisconsin-Madison, Indiana University, and the University of Guelph. Given the established provenance of the statements, no additional pilot testing was conducted prior to data collection.

### Ethical considerations

The study received ethical approval from University of the Aegean’s Research Ethics Committee, approval number 514, on January 15, 2025. All participants provided written informed consent on the Q sort web platform before participation. Data were de-identified and stored securely.

### Statistical analysis

The analysis of the Q sorts, of both the male and female sub-samples, was carried out using KADE v1.3.1, which was selected for its intuitive graphical interface and ease of use.^87^ We employed Principal Components Analysis (PCA) to construct the Q-sort correlation matrix and extract factors that capture shared patterns. To enhance interpretability and reduce dimensionality of data, varimax rotation was subsequently applied, which simplifies the factor structure by ensuring that each statement loads strongly on one factor while loading minimally on others.^88^ This approach facilitates clearer delineation of distinct viewpoints. The ensuing, rotated factor matrix was Q-analysed, a procedure through which the ‘factor arrays’ are computed. Each ‘factor array’ represents the ideal Q-sort of a hypothetical respondent, who in effect would demonstrate ‘perfect agreement’, or a loading of 1, on that particular factor.^86^ Thus, each P-set respondent’s ‘loading’ on the various factors is a measurement of his/her level of agreement with this particular factor/viewpoint. A respondent’s loading on a factor is statistically ‘significant’ when it is sufficiently high to assume that a relationship exists between the respondent and the particular factor. For deciding how many factors to retain for our interpretation, we used the standard statistical criteria for Q studies: all retained factors should have an Eigen value higher than 1, and at least two participants with statistically significant loadings (p<0.05, ‘majority of common variance’ rule applied).^70,78^

## Results

### Sociodemographic characteristics of participants

Of the 30 smallholder farmers included in the study, the majority were male (66.7%, 20/30), while women constituted one third of the sample (33.3%, 10/30). In terms of age, nearly two thirds of the participants (63.3%, 19/30) were below 30 years old, followed by those aged 31 to 40 years (26.7%, 8/30) and 41 to 50 years (10%, 3/30), with no participants above 50 years of age. With respect to educational attainment, the largest proportion held a matric education (33.3%, 10/30), followed by Bachelors and FA/FSc (High School education) holders (23.3% each, 7/30), MA/MSc/MS graduates (16.7%, 5/30), and one participant holding a professional degree (3.3%, 1/30). Regarding village distribution, the majority of participants resided in Pir Adil (53.3%, 16/30), followed by Gadai (20%, 6/30), Choti Zareen and Peer Fateh Shah (10% each, 3/30), and Basti Wadani and Sakhi Sarwar (3.3% each, 1/30) (Table 1).

The results are presented separately for women and men participants to highlight gender-specific patterns in climate change perceptions. For each group, the extracted factors are described along with their defining and distinguishing statements, followed by an interpretation of the shared viewpoints they represent. Consensus statements that reflect common ground across factors are also summarized.

Our first set of results was aimed at identifying gender based distinct factor loadings. Table 2 presents the factor loadings of women farmers to the three distinct factors. A Q-sort with a factor loading of ≥ 0.50 is considered significant and thus defines the particular factor.^70,78^ Table 2 shows the factor loadings for the female sample from which three distinct factors were extracted. Together, the three factors explain 64% of the total variance, and all these factors show high composite reliability.

**Table 2.** Summary of Q-sort factor loading for female sample with defining Q-sorts in bold.

Q-sort	Respondent village	F1	F2	F3
P10	Peer Fateh Shah	**0.79**	-0.1164	0.1286
P8	Pir Adil	**0.7815**	0.1116	0.0364
P5	Pir Adil	**0.717**	0.1699	0.1573
P7	Pir Adil	**0.6277**	0.2324	0.4111
P3	Gadai	-0.1145	**0.7762**	0.1043
P9	Pir Adil	0.1396	**0.7533**	-0.0466
P1	Gadai	0.0787	**0.7357**	0.1295
P2	Gadai	0.2541	**0.677**	0.2197
P6	Pir Adil	0.0715	0.0862	**0.9163**
P4	Pir Adil	0.4004	0.1824	**0.7397**
**No. of Defining Variables**		4	4	2
**% Explained Variance**		24	23	17
**Composite Reliability**		0.941	0.941	0.889

Our data showed 5 distinct factors for men farmers (Table 3). These 5 factors account for 67% of the total variance. All 5 factors have high composite reliability scores, meaning that the perspectives identified are internally consistent and replicable across respondents. These high values reinforce the robustness of the factor interpretations and support the methodological soundness of the analysis.^89^

**Table 3.** Summary of Q-sort factor loading for male sample with defining Q-sorts in bold.

Q-sort	Respondent village	F1	F2	F3	F4	F5
P18	Sakhi Sarwar	**0.8816**	-0.0213	0.1856	0.0278	0.1468
P28	Gadai	**0.7675**	0.0323	0.1373	0.0161	0.0347
P30	Peer Fateh Shah	**0.6680**	0.0766	-0.2051	-0.1041	0.3191
P19	Choti Zareen	0.4617	-0.3728	0.4490	0.3919	0.0098
P13	Choti Zareen	0.0323	**0.7212**	-0.0714	0.0397	0.0436
P26	Peer Fateh Shah	-0.0312	**0.7127**	0.2025	0.0455	-0.2155
P16	Pir Adil	0.2806	**0.6944**	0.1741	0.1323	-0.0362
P23	Pir Adil	-0.2321	**0.6293**	-0.0200	0.2040	0.1310
P20	Choti Zareen	0.5538	0.5714	0.1278	0.2694	0.0586
P24	Pir Adil	-0.0788	-0.0176	**0.9119**	-0.0293	0.0328
P14	Pir Adil	0.1447	0.224	**0.7861**	0.0943	0.0450
P22	Pir Adil	0.1870	-0.0438	**0.5736**	0.0009	0.4133
P11	Pir Adil	-0.4291	-0.2124	**-0.5182**	-0.1873	-0.0269
P17	Pir Adil	-0.1815	0.000	0.0604	**0.8073**	-0.0787
P15	Basti Wadani	0.0628	0.1738	0.0196	**0.6226**	-0.0581
P21	Gadai	0.1780	0.4665	-0.0957	**0.5857**	0.2195
P29	Gadai	0.2410	0.3834	0.2217	0.4949	0.1289
P25	Pir Adil	0.1208	-0.0374	-0.0570	0.0352	**0.7512**
P27	Pir Adil	0.3060	-0.0326	0.1805	0.0828	**0.6355**
P12	Pir Adil	-0.1372	0.1660	0.3679	-0.2790	**0.5832**
**No. of Defining Q-sorts**		3	4	4	3	3
**% Explained Variance**		15	15	13	10	9
**Composite Reliability**		0.923	0.941	0.941	0.923	0.923

### Factors prioritized by women farmers

#### Theme 1: The pragmatist

Factor FW1 is distinguished by its strong emphasis on personal responsibility for climate change, recognizing that certain everyday actions (St. 9) contribute to the problem. Responsibility is not placed solely on urban residents (St. 13) with their cars and industries (St. 14), but also on rural communities through machine-intensive agricultural practices (Sts. 7, 8, 10). While aligning with other perspectives in acknowledging that CC and global warming are occurring (Sts. 6 and 3), Factor 1 diverges in its assessment of current impacts, rejecting the view that local precipitation is declining (St. 5), that CC has reduced product quality (St. 32), and that it has increased management burdens or costs (St. 35). For this group, CC-though real-can be managed through existing mechanisms, with no major detrimental effects anticipated, thus requiring no significant changes (Sts. 38, 42, 43, and 46; see also St. 22). This outlook explains the rejection of specialized forecasts and advisory services (St. 13), reliance on divine intervention (St. 2), and belief in technological solutions (St. 23): from this perspective, neither technology, state intervention, nor supernatural aid is needed to address an issue that is not perceived as a pressing problem. See Table S3 for more details in the supplementary file.

#### Theme 2: The blameless fatalists

In the discourse of Factor FW2 we find pessimistic views about CC. Climate change is happening right now (Sts. 3, 5 & 6), its negative economic effects are already felt (St. 34), and it will mainly hit at home, in the rural areas (Sts. 19 & 21). For this factor, rural communities and farmers have no responsibility for the climate crisis. The farmers’ ‘*machine-intensive agricultural activities*’ (St. 7) and the ‘*intensive use of fertilizers’* (St. 10) are not considered as contributing to CC, while FW2 is mute on the issue of personal responsibility (St. 9). The main culprits are the city dwellers (St. 13), with their cars and industries (St. 14). For this Factor, the future seems bleak: technological progress and fixes will not suffice to turn the tide (St. 23 & 42), adaptation is impossible (St. 41) and the return to normality a hollow dream (St. 22). For FW2, the climate crisis is ultimately beyond humans control, and ‘*it is a matter entrusted to God, and we have nothing to do here’* (St. 1 - a point corroborated by the very weak agreement that ‘*climate change is mainly caused by human activities/actions’* (St. 4)), and only He knows who will be spared (or not) from this climatic Armageddon (St. 2). See Table S4 for more details in the supplementary file.

#### Theme 3: The over-optimistic faithful

In Factor FW3, we find the inverse image of Factor FW2 as long as the causes of CC are concerned. This Factor’s viewpoint demonstrates the strongest possible agreement that ‘*climate change is mainly caused by human activities/actions’* (St. 4; see also Sts. 11, 8, 12, 14 and - to a lesser extent - 7), naturally coupled with the strongest possible rejection of the idea that ‘*CC is a matter entrusted to God, and we have nothing to do here’* (St. 1). FW3 stands out for its rejection of the CC- related negative effects, both current and future, in full contrast with the other two female factors. In FW3’s discourse, CC does not ‘*increase crop diseases and pests’* (St. 27), nor does it ‘*decreases soil fertility and increases soil erosion*’ (St. 31), nor does it ‘*decreases crop yield in our area’* (St. 26). Accordingly, no changes in cultivating practices have occurred (St. 30). Similarly, any future negative effects are also dismissed. FW3 disagrees that ‘*extreme weather events will become more common in the future due to CC’* (St. 20) or that ‘*simultaneous crop failure’* is probable (St. 25). Thus, it is not concerned ‘*about the potentially negative impacts of climate change on my own agriculture’* (St. 16). Having such a dismissing attitude towards CC potential impacts, may explain why FW3 also rejects any measures designed/aiming to tackle/manage CC (see Sts. 23, 46, 48 & 41): similarly, to what we have argued for FW1, why should anyone advocate technological and policy interventions for a non-problem? Nevertheless, there exists another explanation: Factor’s FW3 strong, and distinctive, conviction on God’s protection against CC (St. 2), a belief that naturally renders redundant any human-inspired techno-policy fixes. See Table S5 for more details in the supplementary file.

### Factors prioritized by men farmers

#### Theme 1: The faithful rejector

Factor FM1 is primarily set apart by its rejection of CC current occurrence. This is the only male factor agreeing, and strongly so, that ‘*climate change is still not happening; it may occur decades into the future’* (St. 3). Albeit espousing the general consensus on the anthropogenic causes of CC, such as fossil fuels’ consumption and emissions (St. 12 & 11), and noticing an increase in temperature over time (St. 6), FM1 is characterized by a neutral stance regarding almost every statement relating to CC’s consequences (Sts. 15-24) and effects (Sts. 25-37), arguably due to their rejection of the *current* CC crisis. Current adaptive measures are similarly rejected (Sts. 38-46), with FM1 being confident that ‘*climate change will be solved and we will return to normal conditions in the future*’ (St. 22), albeit strongly, and distinctively, disagreeing that ‘*with our current infrastructure we can cope with the [CC] changes*’ (St. 47, reversed) or that ‘*the impacts of climate change on agriculture are manageable*’ (St. 48). How is this conundrum to be explained, i.e. maintaining that (future) CC is unmanageable and potentially distractive and at the same time believing that things will work out just fine (St. 22)? The answer lays in FM1’s strong belief on an omnipotent and benevolent God who will sort out CC’s ramifications, both generally and in particular. For a Factor strongly believing that ‘*climate change is a matter entrusted to God, and we have nothing to do here’* (St. 1) and that ‘*God will protect my farm household from climate change*’ (St. 2), any human interventions and actions are unnecessary and ultimately redundant. See Table S6 for more details in the supplementary file.

#### Theme 2: The pessimist

Factor FM2 views CC as already occurring (St. 3), affecting both urban and rural areas (Sts. 21, 19), with clear local impacts such as rising temperatures (St. 6), reduced crop yields (St. 26), declining precipitation (St. 5), more frequent extreme weather (St. 15), and worsening soil fertility and erosion (St. 31). Rejecting the idea that CC is solely ‘*entrusted to God*’ (St. 1, reversed), FM2 recognizes rural contributions to CC through machine-intensive agriculture (St. 7) while denying sole urban responsibility (St. 13). Distinctively pessimistic, FM2 anticipates more frequent extreme events (St. 20), rejects the notion that CC will be solved and conditions will return to normal (St. 22), doubts divine protection (St. 2), and remains uncertain about adaptation feasibility (St. 41). It also sees current skill-building initiatives as insufficient (St. 38) and dismisses the potential of additional resources (St. 46) or new technologies (St. 42) to significantly improve coping capacity. See Table S7 for more details in the supplementary file.

#### Theme 3: The faithful pollyanna

Factor FM3 is characterized by a lack of concern about the negative impacts of CC on personal agriculture (St. 16), despite recognizing its anthropogenic nature (St. 4) and emphasizing agricultural/rural causes (Sts. 8, 7) over industrial/urban ones (Sts. 11, 14, 13). It notes reduced precipitation (St. 5), increased animal mortality (St. 37), and more crop diseases and pests (St. 27), addressing these through water and soil conservation (St. 45), but does not link them directly to CC. Neutral on crop yield impacts (St. 26) and dismissive of connections to poverty, food security, and resource depletion (Sts. 29, 28, 18, 17, 24), FM 3 is uncertain about adaptation feasibility (St. 41) or a return to normally (St. 22) and skeptical of institutional, resource-based, or technological solutions (Sts. 38, 47, 46, 39, 42, 23), instead relying on divine will and protection (Sts. 1, 2). See Table S8 for more details in the supplementary file.

#### Theme 4: The unflappable agnostic

Factor FM4 recognises that CC is occurring and is primarily anthropogenic, citing causes such as conversion of forests to farmland (St. 8), fossil fuel use (St. 12), and emissions from industry and transport (Sts. 11, 14). It rejects the notion that CC will affect only distant locations, acknowledging local impacts (St. 19), yet assigns mostly neutral or negative scores to current effect statements, suggesting a perception that present-day consequences are limited. FM4 remains uncertain about humanity’s ability to address CC, giving neutral ratings to statements on adaptation feasibility (St. 41), technological solutions (St. 23), and a return to normal conditions (St. 22). What sets FM4 apart is its optimism about agriculture’s capacity to cope with CC: it agrees that impacts on agriculture are manageable (St. 48) and strongly rejects the view that CC is solely ‘*entrusted to God*’ (St. 1). Instead, it emphasises human agency, acknowledging current infrastructural shortcomings (St. 47) and inadequate funding (St. 40), but supporting the potential of emerging technologies (St. 42), agro-meteorological forecasts, and targeted government support (St. 46) to improve adaptation. This pragmatic orientation contrasts with more fatalistic or passive perspectives on other factors. Full distinguishing and consensus statements for Factor 4 are provided in Supplementary Table S9.

#### Theme 5: The economically worried, techno-faithful

Factor FM5 views CC as already happening (St. 3) but holds ambivalent views on its causes. It slightly disagrees that CC is mainly anthropogenic (St. 4) while strongly agreeing it is a matter ‘*entrusted to God*’ (St. 1). It rejects some causes such as cars and industries (St. 14), intensive fertiliser use (St. 10), and deforestation for agriculture (St. 8), yet agrees that fossil fuel use for electricity (St. 12) and industrial emissions (St. 11) contribute. These inconsistencies suggest a mixed or incomplete understanding of CC causation. Economically, FM 5 stands out for its concern over CC’s impact on farm viability, citing direct experiences of crop loss from heat (St. 36) and income decline (St. 34). This factor expresses high personal concern (St. 16), reports crop diversification (St. 44) and has considered migration (St. 43). However, it does not believe adaptation is possible (St. 41) or that agricultural impacts are manageable (St. 48) and is sceptical of existing or expanded measures to enhance adaptation (Sts. 38, 40, 42, 46, 47). While expecting divine protection (St. 2), it also places some hope in technological progress (St. 23) and eventual return to normal conditions (St. 22). Detailed results for Factor 5 are available in Supplementary Table S10.

### Consensus statements

Both men and women farmers showed consensus on the experiential observation that local temperatures have risen over time (St. 6) and on attributing climate change to anthropogenic causes, particularly the increased use of fossil fuels for electricity production (St. 12). This shared understanding indicates that both men and women recognise human-induced climate change. However, the female sample demonstrated a broader, more multidimensional consensus profile. Women collectively acknowledged changes in rainfall patterns (St. 17), increased animal mortality linked to disease and feed shortages (St. 37), and potential disruptions to food availability (St. 28), elements that were absent from male consensus.

Women's consensus also highlighted systemic and institutional constraints to adaptation, including insufficient and untimely allocation of funds (St. 40), inadequate infrastructure (St. 47), and limited institutional support to enable coping (Sts. 46, 48). In contrast, the men sample reached consensus on fewer statements beyond Sts. 6 and 12, with no shared recognition of these broader livelihood and adaptation concerns. Men sample collectively rejected migration as an adaptation strategy (St. 43) and expressed minimal alignment on other impacts or barriers. The full list of consensus statements and associated scores is provided in Supplementary Tables S11 and S12.

## Discussion

The present study, through decolonial and feminist prims, examines how farmers in Pakistan perceive climate change, with particular attention to how gender shapes these perceptions across causes, consequences, and adaptation pathways. Using cross-sectional Q methodology, we identified distinct perspectives among women and men farmers that show not just differing environmental experiences but also structural inequalities which are historically rooted in gendered divisions of labour, resource access, and decision-making authority. We found that women farmers link climate change to deteriorating food security, infrastructure deficits, and health vulnerabilities which directly exacerbate structural inequalities, increasing their daily labor, care burdens, and vulnerability to violence.^90,91^ Women farmers also frame climate change as an immediate and embodied threat to household survival. While men farmers acknowledge environmental shifts such as rising temperatures and erratic rainfall, they more frequently lean toward technological optimism and market-oriented responses, without interrogating the structural constraints that limit adaptive capacity. Men’s preference for technological solutions demonstrates a broader tendency to prioritize technical fixes over structural transformation. This approach not only sidelines feminist values of justice in climate governance but may also reduce willingness to engage in meaningful climate action, as techno-optimism has been shown to lower the probability of contributing to climate mitigation efforts by nearly 19%.^92,93^ Technological optimism in climate policy and discourse is a reflection of patriarchal systems that encourage climate isolationism. It concentrates solutions in the hands of privileged white men and minimizes the socio-economic innovations.^94^ The divergence in perspectives is a result of systematic epistemic asymmetries between men and women in rural agrarian communities, where climate change is experienced and interpreted through the lens of unequal access to land, information, and institutional support.

Women farmers in our study reported concern about limited infrastructure, inadequate funding from the government institutions, and deteriorating food and animal health, all of which directly affect their physical wellbeing and adaptive capacity. Our findings are consistent with previous studies, where women reported- their adaptive capacity is influenced by environmental stressors, socio-economic and gender inequalities that restrict their access to resources, healthcare, and education.^9,95–98^ Women’s perspectives in our study also showed concern over food insecurity and lack of institutional support. Food insecurity acts as a primary driver of maternal morbidity, mental health challenges, and heat-related illnesses among pregnant women and, more broadly, within climate-exposed rural communities.^99,100^ In Uganda, Peru and the United States, extreme weather has caused severe malnutrition and health crises among women. Their socioeconomic and political exclusion further exacerbates it with restricted access to resources, clean water, education and food security. As a result, women experience worse health outcomes and diseases, including eclampsia, gynaecological cancers, and preterm birth.^101–103^ Climate-driven deterioration of diet quality and the erosion of sustainable dietary patterns can also reduce women’s cardiovascular health. Research shows that strengthening local food systems, improving access to diverse plant-based foods, and promoting women-centered nutrition education could help preserve the cardioprotective benefits of healthy diets.^104^

The presence of fatalistic beliefs in our findings indicates the influence of religious worldviews in shaping interpretations of environmental change. Kabir and colleagues label them as ‘*Theists farmers*’ because of their religious beliefs.^81^ In many rural communities, climate-related disasters are viewed as punishments for human sins. Similar findings have been documented in South Asia, where climate change is often construed as divinely ordained.^82^ In such circumstances, the motivation to pursue adaptive measures is significantly reduced, as human interventions are seen as unnecessary.^105,106^ Male perspectives in our study showed greater diversity, including strongly optimistic and economically motivated factors that placed faith in technological solutions, or otherwise referred as techno-masculine,^94^ and expressed readiness to diversify livelihoods.^59^ However, these narratives were often decoupled from recognition of structural barriers, indicating that men’s adaptation framings tend to be more market oriented. This is consistent with how administrators, scientists, and policymakers tend to prioritise engineering solutions, sectoral adaptation, and economic returns over the social and structural transformations.^107^ In contrast, women’s factors consistently tied adaptation potential to collective, system-level changes, echoing the “community frame” approach,^108^ which integrates individual action within broader institutional and policy contexts.

From a health perspective, the women’s consensus statements (emphasising infrastructure deficits, funding gaps, and deterioration of agricultural health) align with evidence linking environmental shocks to maternal and child health vulnerabilities in climate-exposed rural populations.^109,110^ Several studies from South Asia and beyond have documented people's perspectives about the interconnected pathways through which climate exposure undermine long-term health outcomes.^111–118^ Extreme weather events like floods and droughts destroy crop yields and livestock, leading to acute food insecurity, undernutrition and micronutrient deficiencies that increase the risk of low birth weight and stunting.^119^ Heat stress and high humidity further exacerbate these risks as pregnant women are more susceptible to heat-induced complications like preeclampsia, gestational diabetes, and preterm birth due to compromised thermoregulation.^120–122^ Climate-driven shifts in vector-borne and waterborne diseases, including malaria and cholera, disproportionately affect mothers and children in areas with poor sanitation infrastructure, increasing rates of acute respiratory and diarrheal illness.^123^ These biological stressors are compounded by socioeconomic disruption, where displaced families lose access to essential antenatal and postnatal care, trapping them in a cycle of vulnerability that persists across generations.

Women farmers’ broader consensus profile reflects a multidimensional understanding of climate impacts, linking ecological changes to social and infrastructural constraints.^1,33^ Their emphasis on rainfall shifts, livestock health, and food availability aligns with decolonial arguments that centre lived experience over externally imposed indicators, especially in postcolonial agrarian systems where structural barriers (such as inadequate infrastructure and inequitable resource allocation) are rooted in historical power relations.^5,8^ Men’s consensus statements were narrower, recognising temperature rise and fossil fuel use but overlooking wider livelihood impacts and adaptation barriers. Both men and women farmers’ rejection of migration as an adaptation strategy may reflect socio-cultural norms but also reveals how structural constraints limit perceived options,^4^ making migration less a free choice than a forced response to structural and cultural restrictions, lack of assets, and barriers that disproportionately prevent female farmers from accessing external opportunities.^124^ The other possible factors, such as connection to land, belongings, culture, and religion, as well as familial ties, restrict farmers’ willingness to migrate.^125,126^ From a decolonial lens, such selective framings risk individualising adaptation and obscuring the systemic inequities that underpin vulnerability in rural contexts.^5,127^

### Limitations

To our knowledge, this is the first study to apply Q methodology in Pakistan to examine gendered perspectives on climate change with an explicit focus on implications for women’s health. Several limitations should be noted, and we discuss them here with particular attention to the health-focused readership of this journal. First, this study measures perceptions and subjective framings of climate change. It does not measure health outcomes. The connections we draw between the women’s factors and health risks such as vulnerability, malnutrition, and heat stress are interpretive. They are grounded in an established literature on climate, gender, and health in South Asia, but they are not derived from clinical, anthropometric, or epidemiological data collected from the same participants. We therefore cannot quantify the extent to which the women in our sample have experienced the health risks their viewpoints point toward. Future work that combines Q methodology with concurrent health measurement would allow the perception-to-outcome pathway to be tested directly rather than inferred.

Second, the gender distribution of the sample is uneven, with 10 women and 20 men. This reflects the cultural constraints on women’s participation in public research in rural South Punjab, which we describe in the Methods section. A sample of this size is appropriate for Q methodology, where the analytic goal is to surface the range of viewpoints rather than to achieve population representativeness. However, the smaller female sub-sample means that the three women’s factors rest on a thinner base of loading participants than the five men’s factors, and the stability of the women’s solution should be interpreted with this in mind. The women who did take part were also, by the fact of their participation, those whose mobility and household circumstances permitted it. The perspectives of the most structurally constrained women in the region are likely to be under-represented, and these are precisely the sub-groups whose health is most acutely shaped by climate stress. Future studies in this setting should consider targeted recruitment strategies and home-based data collection to reach them.

Third, the study was conducted in a single district, Dera Ghazi Khan, in southern Punjab. While the four study villages offer socio-economic and geographic diversity within this context, the findings remain specific to rural, agrarian, flood- and heat-exposed communities in southern Punjab. Transferability to other cultural, agro-ecological, or livelihood settings in Pakistan and the wider region should not be assumed. Comparative Q studies across districts and provinces would help establish which framings are locally specific and which travel more widely.

Fourth, the reliance on self-reported perceptions may introduce social desirability bias, particularly on sensitive topics such as religious belief, migration intentions, and trust in government institutions. We took steps to reduce this by framing the Q-sorting as an exercise in personal viewpoint rather than factual knowledge, and by conducting sessions individually and face-to-face. Some residual bias is likely to remain. In addition, although the 48 statements in the Q-set were drawn from previously validated sources and translated and back-translated between English and Urdu, the eligibility and briefing guide was not independently pilot-tested in the local context. This may have introduced some variability in how specific statements were interpreted by participants with different educational backgrounds. The cross-sectional design also captures perceptions at a single point in time and cannot speak to how these framings evolve across seasons or as policy and health system conditions change. Longitudinal designs that track perception shifts and link them to measurable health and livelihood outcomes would represent an important next step.

### Policy implications for women farmers

We identify four areas where interventions can strengthen women farmers’ ability to manage climate-related health risks in rural Pakistan: knowledge and skills, social networks, financial resources, and access to infrastructure and services.

Women farmers with at least a basic level of education, information and climate-related knowledge are more likely to improve their health outcomes, respond effectively, reduce vulnerability to climate shocks, and diversify their crops.^128,129^ With education, women can adapt to flood-resistant practices, manage household nutrition, and prevent heat-related health risks such as stunting, low birth weight, and micronutrient deficiencies. We insist that the Lady Health Worker (LHW) Programme must be used to deliver climate and health knowledge at the household level. When equipped with basic training and digital tools, these workers can help translate early warning information into health-related decisions without challenging any social norms.^130^ This is particularly important for pregnant women who are more vulnerable during heatwaves and flood events. Therefore, strengthening human capital is an important element in improving adaptive capacity and protecting health.

Research also shows that women farmers who participate in community or farmer groups are less vulnerable to food insecurity and related health risks.^98^ Farmer groups are a form of social capital, which is an advantage for women farmers in rural areas because they contribute to access to shared resources, information exchange, and government support. In rural Pakistan, where women’s mobility is restricted, these groups can provide an important space for interaction and collective learning. We recommend forming these groups and sharing information within these groups, which will allow women farmers to improve their knowledge, child health, and access to healthcare services. Similarly, these groups can strengthen relationships between women farmers, extension services, and public institutions, making it easier to access support programmes.

Women with low income and limited savings are more vulnerable to food insecurity and poor health outcomes. Women who are unable to absorb production losses from floods or droughts are more likely to rely on informal credit or remittances, which are usually insufficient during periods of crisis. Limited financial resources restrict the ability of households to purchase nutritious food, access clean water, and seek healthcare when needed. This increases the risk of malnutrition and untreated illness. Social protection mechanisms and direct cash transfers, such as the Benazir Income Support Programme (BISP), can reduce this vulnerability by enabling women farmers to meet basic needs and invest in both agricultural resilience and health.^131,132^

Access to basic infrastructure such as roads, irrigation systems, clean water, and health facilities directly affects the ability of women farmers to respond to environmental shocks. Women farmers living in areas with limited infrastructure and phones face greater barriers to accessing healthcare, particularly during extreme events such as floods and heatwaves.^133,134^ This condition is particularly important for maternal and child health, as timely access to services is required to prevent the impacts of heat stress, waterborne diseases, and food insecurity. Similarly, use of digital technologies (SMS alerts and early warning alerts) can help women farmers prepare for extreme weather events, despite mobility constraints, improve access to information and markets and enhance productivity^135^

## Conclusion

The gendered framings of climate change among rural Pakistani farmers are distinct and structurally embedded, with direct implications for agricultural resilience and population health. Women farmers articulate climate change as a multidimensional crisis encompassing environmental degradation, food and livestock insecurity, infrastructural deficits, and heightened health risks. These framings reveal an acute awareness of systemic barriers that constrain adaptive capacity and exacerbate vulnerability. In contrast, men’s perspectives are more individualised, economically driven, and often reliant on technological optimism, with less consistent recognition of structural determinants. The evidence makes clear that climate adaptation policies must transcend generic, technology-centred solutions and instead prioritise integrated, gender-responsive strategies that address the structural determinants of vulnerability. For women farmers, resilience is contingent on systemic investment in infrastructure, equitable access to resources, and institutional reforms that dismantle barriers to participation in climate governance. Without such structural transformation, adaptation efforts will remain partial, perpetuating existing inequalities and undermining both agricultural sustainability and public health in climate-exposed rural contexts. Specifically for our context, in rural Pakistan, where climate change intersects with gendered divisions of labour and health inequities, equitable adaptation is inseparable from broader struggles for social justice. Embedding adaptation in participatory, community-driven processes offers the most promising route toward climate resilience that safeguards both livelihoods and health.

## Supplemental material

Supplemental material - “When the climate changes, we feel it first”: Gendered perceptions of environmental change and women’s health in Pakistan Supplemental Material for “When the climate changes, we feel it first”: Gendered perceptions of environmental change and women’s health in Pakistan by Abdul Rehman Nawaz, Iosif Botetzagias, Gulnaz Anjum in Women’s Health

Supplemental material - “When the climate changes, we feel it first”: Gendered perceptions of environmental change and women’s health in Pakistan Supplemental Material for “When the climate changes, we feel it first”: Gendered perceptions of environmental change and women’s health in Pakistan by Abdul Rehman Nawaz, Iosif Botetzagias, Gulnaz Anjum in Women’s Health

## Data Availability

We have provided supplemental information and additional details for this paper as open material along with the submission. Additional anonymized data will be provided upon request.*

## Note

1 QTIP (Q Methodology Data Analysis and Visualization Platform). Available at: https://qtip.geography.wisc.edu/#/faq (Accessed: May 17, 2025).
