# Supplementary material for: “When the climate changes, we feel it first”: Gendered perceptions of environmental change and women’s health in Pakistan
Source: Womens Health (Lond). 2026 Jul 7;22:17455057261455288. doi: 10.1177/17455057261455288 (PMC13342432; doi:10.1177/17455057261455288)
Supplement: Supplemental material - “When the climate changes, we feel it first”: Gendered perceptions of environmental change and women’s health in Pakistan [file sj-pdf-1-whe-10.1177_17455057261455288.pdf]

Supplementary Material

Study site

For the data collection, we have selected the flood-prone region of Dera Ghazi Khan (DGK) district in southern Punjab, Pakistan. Between 1950 and 2014, DGK witnessed more than 25 major floods, indicating its vulnerability to seasonal and flash flooding. Within Punjab, the districts of Mianwali, Dera Ghazi Khan, and Rajanpur stand out as some of the most severely affected areas, where flash floods have repeatedly disrupted lives, livelihoods, and local infrastructure (Ahmad et al., 2021). Specifically, in Dera Ghazi Khan, the 2022 floods damaged 342 villages, flooded 80 union councils, and directly impacted nearly 699502 people (Saleem et al., 2023). In June 2025, the Pakistan Meteorological Department (PMD) issued an advisory warning that heavy monsoon rains could trigger flash floods in DGK's hill torrents which led to early relocations and escalated emergency preparedness in the district (OCHA, 2025; The Express Tribune, 2025).

Dera Ghazi Khan is located along the western bank of the Indus River and has experienced recurrent climate-induced events (Birmani, 2022). Geographically, DGK district is situated between the rugged slopes of the Koh-Sulaiman Mountain Range in the west and the expansive Indus River plains in the east. These torrents, flowing down from the Koh-Sulaiman hills, often intensify during the monsoon season and cause sudden and destructive flash floods in downstream communities. Agriculture, livestock rearing, and poultry farming are the major livelihood activities in DGK. Wheat, cotton, sugarcane, and rice are widely cultivated crops. Orchard farming, especially mangoes and citrus, has also seen notable growth (Ahmad & Ma, 2020). Livestock, including cattle, goats, sheep, and poultry, contributes substantially to household income and dietary needs. However, these livelihoods activities are highly sensitive to climate-related events; as they have shown significant reduction in household resilience and increased livelihood vulnerability among small livestock herders (Faisal et al., 2021).

A brief overview of Q methodology

Q methodology was introduced by psychologist William Stephenson in the 1930s to identify shared patterns of thought among participants (Stephenson, 1935). It is hybrid (qualitative and quantitative) research approach and it is used to systematically investigate human subjectivity, particularly individuals' viewpoints, beliefs, and attitudes on any given problem (Robbins, 2009). Unlike traditional statistical methods, so-called R, which are primarily interested in determining the factors affecting (and/or the prominence of) a *particular* opinion, Q methodology seeks to demonstrate holistically the *set* of opinions on a topic which exist in a population, and to identify how these sets of opinions differ to one another. Thus, Q methodology can be said to be 'gestaltist' (Du Plessis, 2005).

A summary of Q methodology's distinctive advantages is provided in **Table S1**).

Table S1: Advantages of Q Methodology

**No.**	**Advantages of Q**	**Description**	**Reference**
1	Captures Operant Subjectivity	Operant subjectivity refers to subjectivity that is revealed through a participant's active sorting of statements. Q methodology allows participants to express their personal viewpoints by actively engaging in the sorting of statements based on their own understanding and lived experiences.	(Brown, 1980; Yang, 2016)
2	Reveals Shared Perspectives	By applying factor analysis to the sorting patterns, Q methodology identifies clusters of individuals who share similar perspectives.	(Tashakkori et al., 2010)
3	Self-referential Sorting	The sorting process in Q is guided entirely by the participant's own frame of reference, allowing them to make meaning from the statements.	(Brown, 1980; Watts & Stenner, 2012)
4	Mixed-Method Approach	Q methodology combines the depth of qualitative interpretation with the structure of quantitative analysis.	(Ramlo & Newman, 2011)
5	Efficient for Small Samples	Unlike conventional surveys that often rely on large representative samples, Q methodology can produce meaningful results with smaller, purposively selected participants.	(McKeown & Thomas, 2014)
6	Useful for Theory Building	Q is particularly suited to exploratory research, where the aim is to unveil, organize and interpret differing viewpoints.	(Ramlo & Newman, 2011)

Outline of a Q-methodology study

Step 1: Defining the Concourse (Q population)

The establishment of the 'concourse' is the foundational phase of Q and provides the base for identifying and interpreting the subjective viewpoints. The term refers to the full range of possible communications - statements, ideas, beliefs, or arguments - that individuals might express on a given particular topic (Van Exel & De Graaf, 2005). McKeown and Thomas (2014) outline various approaches for developing the Q population, that is the statements which encapsulate as fully as possible the diversity and extent of the concourse. These approaches may be 'naturalistic' (i.e. originating from primary sources such as interviews, group discussions and so on), 'ready-made' (i.e. originating from secondary sources such as academic research, literature and so on), or hybrid. Ultimately, it is up to the researcher to determine which approach to follow, as long as the selected statements capture a diverse range of viewpoints to ensure sufficient variability in the Q sample (McKeown & Thomas, 2014).

In this study we employed a ready-made approach for identifying the concourse, by relying on previously developed and validated statements, primarily due to distance and financial constraints. In particular, we relied on two peer-reviewed articles that used Q methodology to examine farmers' perceptions of climate change and adaptive capacity: Kabir et al. (2024), who investigated the farmers' perception of climate change and their adaptation capacity in Bangladesh, and Zobeidi et al. (2016), who examined the perception of Iranian farmers aimed at climate change and food insecurity. The original set of 76 statements extracted from these studies (the Q-population for our study) were categorized into five thematic areas, following (Kabir et al., 2024). : (i) causal beliefs about climate change, (ii) causes of climate change, (iii) consequences/perceived risks of climate change, (iv) impacts of climate change, and (v) adaptation to climate change.

Step 2: Development of Q set

The next step involves constructing the Q set, deciding which statements from those in the broader pool of the Q population will be rank-ordered by the participants of a Q-study (Taheri et al., 2020). There is no fixed rule on the number of statements to be included in a Q set, with suggestions ranging from as low as 40 to as high as 90 (Eeten, 1999; Damio, 2016). Watts and Stenner (2012). Rather the emphasis is on ensuring that the selected Q set is both comprehensive of the concourse and manageable in size (Brown, 1980), since it is through the ranking of the Q set statements that the participants articulate their subjective viewpoints.

For our study, out of the original 76 statements in the Q population, a total of 48 were retained for inclusion in the final Q set (shown in supplementary Table S2).

Table S2: Q-set's statements by thematic category and number

Thematic category	#	Statement
**Causal belief in climate change**	1	Climate change is a matter entrusted to God, and we have nothing to do here.
	2	God will protect my farm household from climate change.
	3	Climate change is still not happening; it may occur decades into the future.
	4	Climate change is mainly caused by human activities/actions.
	5	It seems to me that precipitation in our area is decreasing over time.
	6	It seems to me that the temperatures in our area warmer than in the past.
**Causes of climate change**	7	Machine-intensive agricultural activities by farmers lead to higher concentrations of atmospheric carbon and thus worsen climate change.
	8	Converting land from forests to agriculture contributes to climate change.
	9	I do things on a daily basis that contribute to the problem of climate change.
	10	Intensive use of fertilizer contributes to climate change.
	11	Smoke from mills/industry/cars is responsible for climate change.
	12	Increased use of fossil fuels such as coal, oil and gas for electricity production are responsible for climate change.
	13	Only Urban residents are accountable for climate change.
	14	Cars and industries cause climate change.
**Consequences/perceived risk of climate change**	15	The frequency of extreme weather events (e.g. frost, hail storms, extreme storms, droughts, floods etc) in recent history has accelerated.
	16	I am very concerned about the potentially negative impacts of climate change on my own agriculture.
	17	Changes in the timing of rainfall in our area are related to climate change.
	18	Depletion of groundwater in our area is a result of climate change.

	19	Climate change will mainly affect areas that are far away from here.
	20	Extreme weather would become more common in the future due to climate change.
	21	Climate change likely affects cities more than rural areas
	22	I believe that climate change will be solved, and we will return to normal conditions in the future.
	23	Climate change will be solved through technological progress.
	24	Climate change is the main environmental issue facing farmers in our area right now.
**Effects of climate change**	25	Climate change raises the probability of simultaneous crop failure.
	26	Climate change decreases crop yield in our area.
	27	The nature of crop diseases and pests in our area is increasing due to climate change.
	28	Climate change can disrupt food availability in our area.
	29	Climate change can increase the level of poverty in our area.
	30	I decreased my cultivation land due to climate change.
	31	Soil fertility is decreasing, and soil erosion is increasing in our area due to climate change.
	32	Climate change has negative impacts on the quality of my products.
	33	Global warming would have a considerable impact on human health in our area.
	34	Climate change is having a negative effect on my income.
	35	The increases in warming cause a greater burden on management and labor as well as input costs.
	36	I have seen a crop or farm destroyed by temperature increases.
	37	Animal mortality in our area is increasing due to a lack of forages and diseases.
**Adaptation to climate change**	38	At present, the extension and advisory activities of local organizations on climate change are not enough to improve the skills of farmers.

	39	Access to agro-meteorological forecasts and advice can help climate change adaptation in agriculture.
	40	Insufficient and untimely allocation of funds hinders adaptation efforts to climate change.
	41	It is possible to adapt to climate change.
	42	Different emerging technologies/tools/devices will allow us to strengthen our adaptation to climate change.
	43	I have thought of migrating and pursuing off-farm activities due to climate change.
	44	I am diversifying into other crops due to climate change.
	45	I use water and soil conservation techniques due to climate change.
	46	With wealth, credit, education, and information provided by the government, it is possible to cope with climate change.
	47	With our current infrastructure we cannot cope with the changes.
	48	I believe that the impacts of climate change on agriculture are manageable.

Step 3: Selection of P Set

In Q methodology, the P set refers to the group of selected participants who perform the Q sorting task. As we have already mentioned, in contrast to conventional R methodology, which seeks statistical generalizability through large, representative samples, Q studies prioritize the identification of the range of subjectivities within a given context (Brown, 1980). Accordingly, participants in Q studies are selected based on purposive and convenience sampling techniques and their capacity to contribute informed and diverse viewpoints (Watts & Stenner, 2012). Q methodology works well with small but strategically chosen samples of typically 20 to 40 participants (Van Exel & De Graaf, 2005).

Step 4: Q Sorting

Following the selection of P set, the fourth and the last step is Q sorting. This entails the ranking by the participants of each statement on a continuum ranging from "*most agree*" to "*most disagree*" (Watts & Stenner, 2012). In our research we employed a nine-points Likert scale, from -4 to +4 respectively, with 0 as the middle point. In Q sorting, the zero value does not necessarily mean neutrality or indifference but rather it ‘operates as a meaningful hub or centre from and around which positive and negative salience, the meaning of the Q-sort and the variability of the distribution, *distend*’ (Watts and Stenner 2012, p.79). Prior to sorting, potential participants were provided with a brief explanation of the purpose of the study and the meaning of each statement, ensuring clarity and minimizing the risk of misinterpretation. After that, a brief eligibility survey was conducted to collect demographic information and assess whether participants met the study’s criteria. Informed consent was obtained before proceeding with the Q-sort

activity. Then, the participants were presented with the statements translated in Urdu and conducted their q-sorting in a quiet and neutral setting to avoid peer influence and allow participants sufficient time to ponder. The sorting process was conducted face-to-face between April 8 and April 24, 2025. This study employed Q-TIP[1], a free, web-based tool developed by researchers from the University of Wisconsin–Madison, Indiana University, and the University of Guelph. Participants used digital tablets to complete the sorting process via Q-TIP, which streamlined the exercise and improved user engagement.

In this study, participants were restricted in the number of items they could place in each Likert-scale category of the Q-sort grid, which produces a U-shaped distribution with fewer statements permitted at the extreme ends and a greater number concentrated near the neutral center (see Figure S2).

Figure S2: Shape of Q sort grid used in the Q sorting, with the number of statements allowed per category in [brackets], and example of a completed Q-sort (by Participant 29)

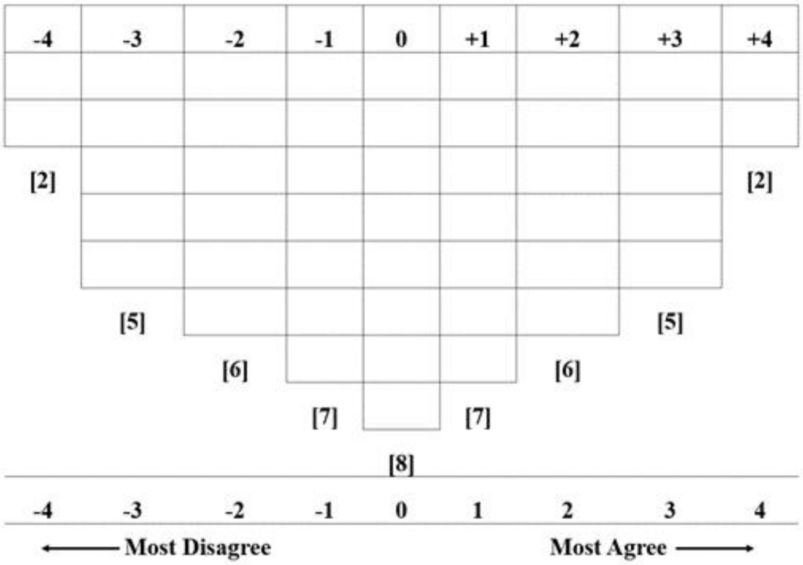

P20

-4	-3	-2	-1	0	1	2	3	4
3	22	19	2	1	9	4	6	8
46	40	23	13	16	10	5	7	11
	43	34	32	26	17	14	12	
	47	36	37	29	21	15	20	
	48	41	38	30	24	18	33	
		42	39	31	25	28		
			45	35	27			
				44				

Source: Authors' own compilation.

[1] *QTIP (Q Methodology Data Analysis and Visualization Platform) is an open-source web application developed by the University of Wisconsin–Madison for analyzing and interpreting Q methodology data. Available at: https://qtip.geography.wisc.edu/#/faq (Accessed: May 17, 2025).*

Factors for Women

Table S3: Most (dis)agreed (±4 & ±3) and distinguishing (*) statements for Factor FW1: Accomplices to an issue which is not (yet) a problem

St No	Statement	FW1	FW2	FW3
7	Machine-intensive agricultural activities by farmers lead to higher concentrations of atmospheric carbon and thus worsen climate change.	4*	-1	2
8	Converting land from forests to agriculture contributes to climate change.	4	3	3
6	It seems to me that the temperatures in our area warmer than in the past.	3	3	2
9	I do things on a daily basis that contribute to the problem of climate change.	3*	0	1
10	Intensive use of fertilizer contributes to climate change.	3	-3	2
12	Increased use of fossil fuels such as coal, oil and gas for electricity production are responsible for climate change.	3	3	3
18	Depletion of groundwater in our area is a result of climate change.	3*	-1	0
5	It seems to me that precipitation in our area is decreasing over time.	1*	4	3
14	Cars and industries cause climate change.	0*	3	3
23	Climate change will be solved through technological progress.	0*	-3	-3
35	The increases in warming cause a greater burden on management and labor as well as input costs.	-1*	1	1
32	Climate change has negative impacts on the quality of my products.	-2*	0	2
39	Access to agro-meteorological forecasts and advice can help climate change adaptation in agriculture.	-2*	0	0
13	Only Urban residents are accountable for climate change.	-2*	3	2
3	Climate change is still not happening; it may occur decades into the future.	-3	-4	-2
38	At present, the extension and advisory activities of local organizations on climate change are not enough to improve the skills of farmers.	-3	1	-1
42	Different emerging technologies/tools/devices will allow us to strengthen our adaptation to climate change.	-3	-3	-2
43	I have thought of migrating and pursuing off-farm activities due to climate change.	-3	-2	0
46	With wealth, credit, education, and information provided by the government, it is possible to cope with climate change.	-3	-2	-3
1	Climate change is a matter entrusted to God, and we have nothing to do here.	-4	4	-4
2	God will protect my farm household from climate change.	-4*	0	3

Table S4: Most (dis)agreed (±4 & ±3) and distinguishing (*) statements for Factor FW2: The blameless fatalists

St No	Statement	FW1	FW2	FW3
5	It seems to me that precipitation in our area is decreasing over time.	1	4	3
1	Climate change is a matter entrusted to God, and we have nothing to do here.	-4	4*	-4
8	Converting land from forests to agriculture contributes to climate change.	4	3	3
6	It seems to me that the temperatures in our area warmer than in the past.	3	3	2
12	Increased use of fossil fuels such as coal, oil and gas for electricity production are responsible for climate change.	3	3	3
13	Only Urban residents are accountable for climate change.	-2	3	2
14	Cars and industries cause climate change.	0	3	3
34	Climate change is having negative effect on my income.	-1	2*	-1
2	God will protect my farm household from climate change.	-4	0*	3
32	Climate change has negative impacts on the quality of my products.	-2	0*	2
7	Machine-intensive agricultural activities by farmers lead to higher concentrations of atmospheric carbon and thus worsen climate change.	4	-1*	2
21	Climate change likely affects cities more than rural areas.	1	-1*	1
10	Intensive use of fertilizer contributes to climate change.	3	-3*	2
42	Different emerging technologies/tools/devices will allow us to strengthen our adaptation to climate change.	-3	-3	-2
19	Climate change will mainly affect areas that are far away from here.	0	-3*	1
23	Climate change will be solved through technological progress.	0	-3	-3
41	It is possible to adapt to climate change.	-1	-3	-4
3	Climate change is still not happening; it may occur decades into the future.	-3	-4	-2
22	I believe that climate change will be solved and we will return to normal conditions in the future.	0	-4*	1

Table S5: Most (dis)agreed (±4 & ±3) and distinguishing (*) statements for Factor FW3: The over-optimistic faithful

St No	Statement	FW1	FW2	FW3
4	Climate change is mainly caused by human activities/actions.	2	1	4*
11	Smoke from mills/industry/cars is responsible for climate change.	1	2	4*
5	It seems to me that precipitation in our area is decreasing over time.	1	4	3
8	Converting land from forests to agriculture contributes to climate change.	4	3	3
12	Increased use of fossil fuels such as coal, oil and gas for electricity production are responsible for climate change.	3	3	3
14	Cars and industries cause climate change.	0	3	3
2	God will protect my farm household from climate change.	-4	0	3*
23	Climate change will be solved through technological progress.	0	-3	-3
32	Climate change has negative impacts on the quality of my products.	-2	0	2*
7	Machine-intensive agricultural activities by farmers lead to higher concentrations of atmospheric carbon and thus worsen climate change.	4	-1	2*
27	The nature of crop diseases and pests in our area is increasing due to climate change.	2	1	-1*
20	Extreme weather would become more common in the future due to climate change.	1	2	-2*
31	Soil fertility is decreasing and soil erosion increasing in our area due to climate change.	0	2	-2*
16	I am very concerned about the potentially negative impacts of climate change on my own agriculture.	1	0	-2*
25	Climate change raises the probability of simultaneous crop failure.	2	2	-2*
26	Climate change decreases crop yield in our area.	2	2	-3*
30	I decreased my cultivation land due to climate change.	0	0	-3*
46	With wealth, credit, education, and information provided by the government, it is possible to cope with climate change.	-3	-2	-3
48	I believe that the impacts of climate change on agriculture are manageable.	-2	-2	-3
1	Climate change is a matter entrusted to God, and we have nothing to do here.	-4	4	-4
41	It is possible to adapt to climate change.	-1	-3	-4

Factors by men farmers

Table S6: Most (dis)agreed (±4 & ±3)and distinguishing (*) statements for Factor FM1: The Faithful Rejector

St No	Statement	FM1	FM2	FM3	FM4	FM5
1	Climate change is a matter entrusted to God, and we have nothing to do here.	4	-4	4	-4	4
2	God will protect my farm household from climate change.	4	-2	4	-1	3
3	Climate change is still not happening; it may occur decades into the future.	3*	-4	-2	-2	-4
6	It seems to me that the temperatures in our area warmer than in the past.	3	4	3	2	2
11	Smoke from mills/industry/cars is responsible for climate change.	3	2	0	4	3
12	Increased use of fossil fuels such as coal, oil and gas for electricity production are responsible for climate change.	3	1	1	3	1
22	I believe that climate change will be solved and we will return to normal conditions in the future.	3	-2	0	0	3
33	Global warming would have a considerable impact on human health in our area.	-2*	2	1	1	1
39	Access to agro-meteorological forecasts and advice can help climate change adaptation in agriculture.	-2*	0	1	2	2
42	Different emerging technologies/tools/devices will allow us to strengthen our adaptation to climate change.	-3	-3	1	3	-1
43	I have thought of migrating and pursuing off-farm activities due to climate change.	-3	-2	-1	-2	0
44	I am diversifying into other crops due to climate change.	-3	-3	0	-1	1
45	I use water and soil conservation techniques due to climate change.	-3*	-1	2	-1	-1
46	With wealth, credit, education, and information provided by the government, it is possible to cope with climate change.	-3	-3	0	3	-1
47	With our current infrastructure we cannot cope with the changes.	-4*	-1	2	3	0
48	I believe that the impacts of climate change on agriculture are manageable.	-4*	-1	1	3	-2

Table S7: Most (dis)agreed (±4 & ±3)and distinguishing (*) statements for Factor FM2: The Pessimist

St No	Statement	FM1	FM2	FM3	FM4	FM5
6	It seems to me that the temperatures in our area warmer than in the past.	3	4	3	2	2
26	Climate change decreases crop yield in our area.	0	4	0	0	2
5	It seems to me that precipitation in our area is decreasing over time.	2	3	3	-2	3
7	Machine-intensive agricultural activities by farmers lead to higher concentrations of atmospheric carbon and thus worsen climate change.	2	3	2	-2	0
15	The frequency of extreme weather events (e.g. frost, hail storms, extreme storms, droughts, floods etc) in recent history has accelerated.	2	3	0	1	-2
20	Extreme weather would become more common in the future due to climate change.	0	3*	2	-1	-2
38	At present, the extension and advisory activities of local organizations on climate change are not enough to improve the skills of farmers.	-2	3*	0	0	-2
31	Soil fertility is decreasing and soil erosion increasing in our area due to climate change.	-1	1*	-1	-3	-3
34	Climate change is having negative effect on my income.	-1	0*	-1	-3	3
21	Climate change likely affects cities more than rural areas.	2	-1*	2	0	1
22	I believe that climate change will be solved and we will return to normal conditions in the future.	3	-2*	0	0	3
18	Depletion of groundwater in our area is a result of climate change.	1	-2*	-4	1	0
13	Only Urban residents are accountable for climate change.	1	-3	-3	1	-4
19	Climate change will mainly affect areas that are far away from here.	0	-3	-3	-4	-1
42	Different emerging technologies/tools/devices will allow us to strengthen our adaptation to climate change.	-3	-3	1	3	-1
44	I am diversifying into other crops due to climate change.	-3	-3	0	-1	1
46	With wealth, credit, education, and information provided by the government, it is possible to cope with climate change.	-3	-3	0	3	-1
1	Climate change is a matter entrusted to God, and we have nothing to do here.	4	-4	4	-4	4
3	Climate change is still not happening; it may occur decades into the future.	3	-4	-2	-2	-4

Table S8: Most (dis)agreed (±4 & ±3) and distinguishing (*) statements for Factor FM3: The faithful Pollyanna

St No	Statement	FM1	FM2	FM3	FM4	FM5
1	Climate change is a matter entrusted to God, and we have nothing to do here.	4	-4	4	-4	4
2	God will protect my farm household from climate change.	4	-2	4	-1	3
4	Climate change is mainly caused by human activities/actions.	1	1	3	2	-1
5	It seems to me that precipitation in our area is decreasing over time.	2	3	3	-2	3
6	It seems to me that the temperatures in our area warmer than in the past.	3	4	3	2	2
8	Converting land from forests to agriculture contributes to climate change.	1	1	3	4	-3
37	Animal mortality in our area is increasing due to a lack of forage and diseases.	-2	1	3	-3	0
45	I use water and soil conservation techniques due to climate change.	-3	-1	2*	-1	-1
11	Smoke from mills/industry/cars is responsible for climate change.	3	2	0*	4	3
14	Cars and industries cause climate change.	2	0	-1*	2	-3
13	Only Urban residents are accountable for climate change.	1	-3	-3	1	-4
17	Changes in the timing of rainfall in our area are related to climate change.	1	-2	-3	2	1
19	Climate change will mainly affect areas that are far away from here.	0	-3	-3	-4	-1
23	Climate change will be solved through technological progress.	1	-2	-3	0	2
29	Climate change can increase the level of poverty in our area.	0	0	-3*	-1	4
16	I am very concerned about the potentially negative impacts of climate change on my own agriculture.	1	2	-4*	1	2
18	Depletion of groundwater in our area is a result of climate change.	1	-2	-4*	1	0

Table S9: Most (dis)agreed (±4 & ±3) and distinguishing (*) statements for Factor FM4: The unflappable agnostic

St No	Statement	FM1	FM2	FM3	FM4	FM5
8	Converting land from forests to agriculture contributes to climate change.	1	1	3	4*	-3
11	Smoke from mills/industry/cars is responsible for climate change.	3	2	0	4	3
12	Increased use of fossil fuels such as coal, oil and gas for electricity production are responsible for climate change.	3	1	1	3	1
42	Different emerging technologies/tools/devices will allow us to strengthen our adaptation to climate change.	-3	-3	1	3	-1
46	With wealth, credit, education, and information provided by the government, it is possible to cope with climate change.	-3	-3	0	3*	-1
47	With our current infrastructure we cannot cope with the changes.	-4	-1	2	3	0
48	I believe that the impacts of climate change on agriculture are manageable.	-4	-1	1	3*	-2
5	It seems to me that precipitation in our area is decreasing over time.	2	3	3	-2*	3
30	I decreased my cultivation land due to climate change.	-1	-1	-2	-3	-2
31	Soil fertility is decreasing and soil erosion increasing in our area due to climate change.	-1	1	-1	-3	-3
34	Climate change is having negative effect on my income.	-1	0	-1	-3	3
35	The increases in warming cause a greater burden on management and labor as well as input costs.	-1	-1	-2	-3	-3
37	Animal mortality in our area is increasing due to a lack of forage and diseases.	-2	1	3	-3	0
1	Climate change is a matter entrusted to God, and we have nothing to do here.	4	-4	4	-4	4
19	Climate change will mainly affect areas that are far away from here.	0	-3	-3	-4	-1

Table S10: Most (dis)agreed (±4 & ±3) and distinguishing (*) statements for Factor FM5: The economically worried, techno-faithfull

St No	Statement	FM1	FM2	FM3	FM4	FM5
1	Climate change is a matter entrusted to God, and we have nothing to do here.	4	-4	4	-4	4
29	Climate change can increase the level of poverty in our area.	0	0	-3	-1	4*
2	God will protect my farm household from climate change.	4	-2	4	-1	3
5	It seems to me that precipitation in our area is decreasing over time.	2	3	3	-2	3
11	Smoke from mills/industry/cars is responsible for climate change.	3	2	0	4	3
22	I believe that climate change will be solved and we will return to normal conditions in the future.	3	-2	0	0	3
34	Climate change is having negative effect on my income.	-1	0	-1	-3	3*
36	I have seen a crop or farm destroyed by temperature increases.	-1	-1	-1	-2	2
37	Animal mortality in our area is increasing due to a lack of forage and diseases.	-2	1	3	-3	0
8	Converting land from forests to agriculture contributes to climate change.	1	1	3	4	-3*
14	Cars and industries cause climate change.	2	0	-1	2	-3*
31	Soil fertility is decreasing and soil erosion increasing in our area due to climate change.	-1	1	-1	-3	-3
32	Climate change has negative impacts on the quality of my products.	-1	0	-1	-1	-3
35	The increases in warming cause a greater burden on management and labor as well as input costs.	-1	-1	-2	-3	-3
3	Climate change is still not happening; it may occur decades into the future.	3	-4	-2	-2	-4
13	Only Urban residents are accountable for climate change.	1	-3	-3	1	-4

Consensus statements

Table S11: Consensus Statements for women farmers

St No	Statement	FW1	FW2	FW3
3	Climate change is still not happening; it may occur decades into the future.	-3	-4	-2
6	It seems to me that the temperatures in our area warmer than in the past.	3	3	2
8	Converting land from forests to agriculture contributes to climate change.	4	3	3
11	Smoke from mills/industry/cars is responsible for climate change.	1	2	4
12	Increased use of fossil fuels such as coal, oil and gas for electricity production are responsible for climate change.	3	3	3
15	The frequency of extreme weather events (e.g. frost, hail storms, extreme storms, droughts, floods etc) in recent history has accelerated.	2	1	0
17	Changes in the timing of rainfall in our area are related to climate change.	1	0	1
24	Climate change is the main environmental issue facing farmers in our area right now.	1	-1	0
28	Climate change can disrupt food availability in our area.	2	0	-1
33	Global warming would have a considerable impact on human health in our area.	0	1	0
35	The increases in warming cause a greater burden on management and labor as well as input costs.	-1	1	1
37	Animal mortality in our area is increasing due to a lack of forage and diseases.	-1	-1	0
39	Access to agro-meteorological forecasts and advice can help climate change adaptation in agriculture.	-2	0	0
40	Insufficient and untimely allocation of funds hinders adaptation efforts to climate change.	-1	1	-1
41	It is possible to adapt to climate change.	-1	-3	-4
42	Different emerging technologies/tools/devices will allow us to strengthen our adaptation to climate change.	-3	-3	-2
44	I am diversifying into other crops due to climate change.	-2	-1	-1
45	I use water and soil conservation techniques due to climate change.	-2	-2	-1
46	With wealth, credit, education, and information provided by the government, it is possible to cope with climate change.	-3	-2	-3
47	With our current infrastructure we cannot cope with the changes.	-1	-2	0
48	I believe that the impacts of climate change on agriculture are manageable.	-2	-2	-3

Table S12: Consensus Statements for men farmers

St No	Statement	FM1	FM2	FM3	FM4	FM5
6	It seems to me that the temperatures in our area warmer than in the past.	3	4	3	2	2
12	Increased use of fossil fuels such as coal, oil and gas for electricity production are responsible for climate change.	3	1	1	3	1
25	Climate change raises the probability of simultaneous crop failure.	0	0	1	1	0
43	I have thought of migrating and pursuing off-farm activities due to climate change.	-3	-2	-1	-2	0
